# Use of Motivational Interviewing Within Home-Based Primary Care: A Gamification Approach

**DOI:** 10.1093/geroni/igaf122.054

**Published:** 2025-12-31

**Authors:** Michelle Mlinac, Angelica Boeve, Tara Afonso, Cynthia Knight, Katie Mendoza, Hannah Bashian, Brian Green, Linda DiLalla

**Affiliations:** VA Boston Healthcare System, Boston, Massachusetts, United States; Minneapolis VA, Minneapolis, Minnesota, United States; VA Boston Health Care System, Boston, Massachusetts, United States; Jamaica Plain VA Medical Center, Jamaica Plain, Massachusetts, United States; VA Boston Health Care System, Boston, Massachusetts, United States; VA Boston Health Care System, Boston, Massachusetts, United States; VA Boston Health Care System, Boston, Massachusetts, United States; VA Boston Health Care System, Boston, Massachusetts, United States

## Abstract

Motivational interviewing (MI) is a well-established strategy for promoting behavior change, but it has been understudied for older adults with complex health conditions. As part of a holistic patient-centered approach to the care of older patients with multi-complexity, such as those served by home-based primary care (HBPC), use of MI could be instrumental in identifying patients’ priorities, strengthening interdisciplinary care, and engaging patients in managing their own healthcare decisions. A multi-pronged approach to training a home-based primary care team in using MI was undertaken to train HBPC providers in practicing MI regularly during patient care. A gamification teaching strategy was utilized to train 24 members of an interprofessional HBPC in use of MI skills. A 90-minute workshop was comprised of a 30-minute didactic and a 60-minute skills-focused game, led by three MI experts within the team. Participants were asked to complete brief pre- and post-session knowledge checks. The number of participants correctly identifying the stages of behavior change and MI microskills increased from 54% and 75% respectively before the training, to 100% on both concepts after the session. Qualitative feedback was obtained from a focus group conducted with team members after the training highlighted collaboration and feeling valued as positive impacts of using MI. Sustainability efforts following the training included having time during team meetings to share of stories about using MI with patients and building in in-home coaching sessions by MI experts within the team to demonstrate MI skills with patients and help coach staff who requested additional learning.

